# Applications of Holographic Augmented Reality in Psychology: Current and Future Perspectives

**DOI:** 10.1192/j.eurpsy.2026.11354

**Published:** 2026-07-31

**Authors:** G. M. Festa, I. S. Lancia

**Affiliations:** 1 Pontifical Faculty of Educational Sciences «AUXILIUM»; 2 Interdisciplinary Institute of Advanced Clinical Training «IACT», Rome, Italy

## Abstract

**Introduction:**

Immersive technologies are opening new frontiers across various areas of psychological practice, both general and clinical. Among them, Holographic Augmented Reality (HAR) enables the three-dimensional projection of images into real space without the need for headsets or glasses, allowing for direct interaction with complex visual content. Already employed in the medical and academic fields, HAR is now emerging as a valuable tool for psychology as well.

For an in-depth exploration, see: Festa, G. M., Lancia, I. S. (2025). *Exploring the Psychological Potential of 3D Holography: from Psychoeducation to Clinical Practice*, *Journal of Behavioral Health and Psychology*, 14(4).

**Objectives:**

This paper aims to provide an overview of the potential applications of HAR in psychological contexts through a review of existing scientific literature. The goal is to highlight how this technology can be integrated into training, prevention, psychoeducation, and clinical treatment practices.

**Methods:**

A review of international literature was conducted concerning the use of HAR in medical, educational, and psychological settings. Scientific articles and pilot project reports on the use of holographic augmented reality in psychological, clinical, and rehabilitative interventions were selected and analyzed.

**Results:**

The evidence collected suggests that HAR can be effectively used in multiple domains:

- Training and psychoeducation, by making abstract concepts more accessible and understandable;
- Stress prevention and management, through immersive and engaging experiences;
- Clinical treatment, in support of cognitive restructuring, emotional regulation, enhancement of social skills, cognitive rehabilitation, and interventions for specific phobic and trauma-related disorders.

Currently, however, the application of HAR in psychology remains limited and is still in an experimental phase.

**Conclusions:**

Holographic Augmented Reality represents a promising technological frontier for psychology. Although its clinical and educational applications are still under investigation, preliminary findings suggest significant potential for implementing innovative psychological interventions. The development of validated tools and the integration with evidence-based methodologies will be essential to ensure the effectiveness and ethical use of HAR in future psychological contexts.

**Disclosure of Interest:**

None Declared

